# CRISPR-Cas9 mediated one-step disabling of pancreatogenesis in pigs

**DOI:** 10.1038/s41598-017-08596-5

**Published:** 2017-09-05

**Authors:** Jun Wu, Marcela Vilarino, Keiichiro Suzuki, Daiji Okamura, Yanina Soledad Bogliotti, Insung Park, Joan Rowe, Bret McNabb, Pablo Juan Ross, Juan Carlos Izpisua Belmonte

**Affiliations:** 1 Salk Institute for Biological Studies, 10010 N. Torrey Pines Rd., La Jolla, CA 92037 USA; 2 Universidad Católica San Antonio de Murcia, Campus de los Jerónimos 135, Guadalupe, 30107 Spain; 3 Department of Animal Science, University of California Davis, One Shields Avenue, Davis, CA 95616 USA; 4 Department of Advanced Bioscience, Graduate School of Agriculture, Kindai University, 3327-204 Nakamachi, Nara, 631-8505 Japan; 5 School of Veterinary Medicine, University of California Davis, One Shields Avenue, Davis, CA 95616 USA

## Abstract

Genome editing using programmable nucleases has revolutionized biomedical research. CRISPR-Cas9 mediated zygote genome editing enables high efficient production of knockout animals suitable for studying development and relevant human diseases. Here we report efficient disabling pancreatogenesis in pig embryos via zygotic co-delivery of Cas9 mRNA and dual sgRNAs targeting the *PDX1* gene, which when combined with chimeric-competent human pluriopotent stem cells may serve as a suitable platform for the xeno-generation of human tissues and organs in pigs.

## Introduction

Interspecies blastocyst complementation combines the chimera-forming capability of donor pluripotent stem cells (PSCs) and organogenesis-disabled hosts, which allows for the enrichment of donor cells in a target organ^1, 2^. This raises an intriguing possibility to generate human organs using easy accessible host animals that are similar to humans in organ-size, physiology, and gestational period^3^. In this regard, large livestock species, e.g. pigs, cattle and sheep, emerge as more relevant host species for humans. Thanks to the rapid development of targeted genome engineering technologies, particularly the CRISPR (Clustered Regularly Interspaced Short Palindromic Repeat) and CRISPR-associated protein 9 (Cas9), targeted gene inactivation in livestock species, once difficult, can now be achieved relatively with ease via zygotic co-delivery of Cas9 mRNA/protein and single-guide RNA (sgRNA)^4, 5^. To date, however, it remains to be demonstrated whether CRISPR-Cas9 based zygotic genome editing can be efficiently harnessed for the generation of organogenesis-disabled large livestock host embryos suitable for interspecies complementation with human PSCs. Here we report the efficient gene disruption in porcine blastocysts, and robust generation of pancreatogenesis disabled E28 pig embryos via zygotic co-injection of Cas9 mRNA and dual sgRNAs targeting the *PDX1* gene.

*Pdx1* plays an essential role in mouse pancreatic development and mice lacking *Pdx1* die within a few days of birth due to the lack of a pancreas^6, 7^. Apancreatic phenotype could also be observed in transgenic pigs with the forced expression of HES1 (hairy and enhancer of split 1) under the *PDX1* promoter^8^. However, to date, it remains unknown whether the function of PDX1 is conserved across distant species and the disruption of *PDX1* gene itself can disable pancreas development in large livestock species. To this end, we first designed and generated a sgRNA (sgRNA1) targeting exon 1 of both pig and cattle *PDX1* genes. Bovine and porcine embryos were produced by *in vitro* fertilization (IVF) and parthenogenesis, respectively, using *in vitro* matured oocytes collected from slaughterhouse derived ovaries. We performed intracytoplasmic co-injection of Cas9 mRNA and PDX1 sgRNA1 into both porcine and bovine zygotes. After injection, embryos were cultured *in vitro* until the blastocyst stage before genomic DNA was extracted and used for embryo genotyping by Sanger sequencing (Supplementary information, Figure S1A). For cattle, among 12 blastocysts, we found two contained wild type sequence at the *PDX1* locus and the remaining 10 (83.3%, 10/12) showed indels among which half (5/10) were mono-allelic and the other half (5/10) were bi-allelic (Supplementary information, Figure S1B). Curiously, most of the indels did not alter the *PDX1* open reading frame and are thus unlikely to result in protein functional disruption (Supplementary information, Figure S1B). For pig we only observed 15% (2/13) of the blastocysts contained monoallelic mutations and no biallelically mutated blastocyst was obtained (Supplementary information, Figure S1C). These results show that, although effective, the use of single sgRNA-guided Cas9 for generating gene knockouts in large livestock species is unpredictable. In addition, it is time-consuming and laborious to identy single sgRNA generated indels, which are often small.

**Figure 1:**
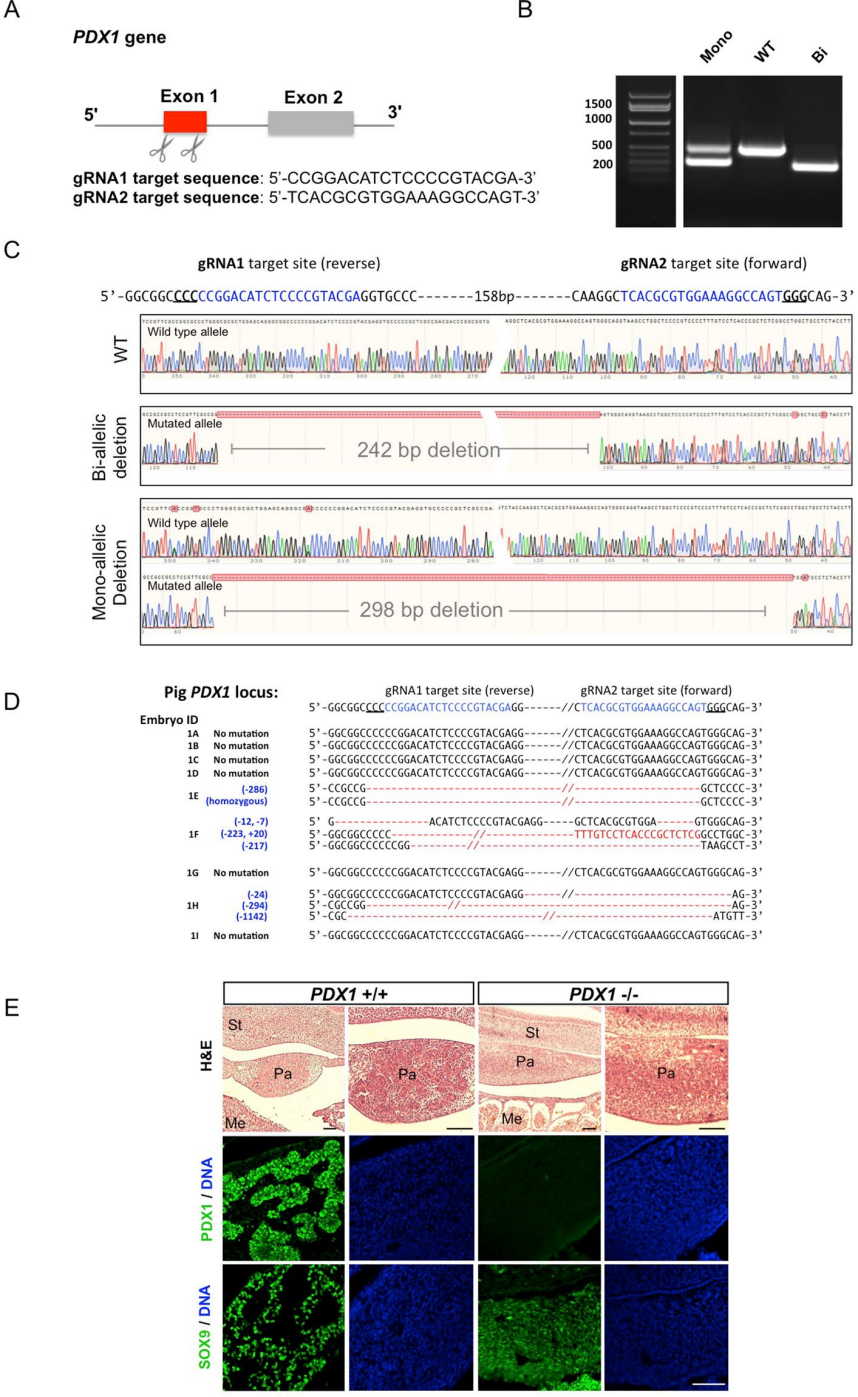
Dual sgRNA-guided Cas9 enables efficient and effective disabling of pancreatogenesis in pigs. (**A**) Schematic diagram of the location and sequences of the two gRNAs designed to target the exon 1 of *PDX1* gene. (**B**) A representative DNA gel image showing PCR products of the target region of a wild type, a bi-allelic and mono-allelic *PDX1* mutant pig blastocyst. Bi, bi-allelic; Mono, mono-allelic. The grouping of gels was cropped out from different parts from the same gel (Supplementary information). (**C**) Sanger sequencing chromatograms of three blastocysts described in Fig. 1B. (**D**) Sanger sequencing results from collected E28 pig embryos. Red dashes and // represent deletions; red letters indicate insertions; insertions (+) or deletions (−) are shown to the left of each allele, e.g. −12, deletion of 12 nucleotides; +20, insertion of 20 nucleotides. Embryo ID was shown on the left. (E) Representative H&E staining (top) and immunofluorescence images (middle and bottom) of the pancreatic primordium from wildtype and *PDX1* ^−/−^ E28 pig embryos. Green, PDX1 and SOX9 immunostaining. Blue, DAPI. St, stomach; Pa, pancreas; Me, mesonephros. Scale bar, 200 μm.

The use of CRISPR-Cas9 system in conjunction with dual sgRNAs has been shown to be a robust method for generating large gene deletions^9, 10^. Next we designed another sgRNA also targeting exon 1 of the pig *PDX1* gene (sgRNA2) (Fig. 1A). The distance between the cut sites of sgRNA1 and sgRNA2 is 204 bp. We co-injected Cas9 mRNA, sgRNA1 and sgRNA2 together into the cytoplasm of pig zygotes followed by *in vitro* culture to blastocysts (Supplementary information, Figure S2A). In contrast to single sgRNA, bi-allelic and mono-allelic mutants generated by dual sgRNAs could be easily identified by simple PCR amplification of the targeted region and DNA gel electrophresis. As shown in Fig. 1B, a ~200 bp deletion could be distingushied from the wild type allele. Sanger sequencing further revealed that bi-allelic mutants had a homozygous 242 bp deletion and the mono-allelic mutant a heterozygous 299 bp deletion (Fig. 1C). Dual sgRNA-guided Cas9 nuclease yielded 59% of mutant blastocysts (13/22) among which 77% (10/13) were mono-allelic and 23% (3/13) bi-allelic, a major improvement over single sgRNA for pig embryos (Supplementary information, Figure S2B). These results demonstrate that the combination of Cas9 and dual-sgRNAs is a powerful platfrom for gene knockout in pig embryos and deletion of a large DNA fragment faciliates mutant screening.

Next, to study whether *PDX1* knockout can disrupt pancreas development in pigs, we performed embryo transfer using gene-edited pig blastocysts generated by dual sgRNA/Cas9 injection into *in vivo* produced zygotes. We co-transferred 18 injected blastocysts together with 5 un-injected blastocysts to a surrogate sow. At E28 we stopped the pregancy and retrieved 9 embryos with apparent normal morphology and body size (Supplementary information, Figure S2C). Genomic DNA was extracted from the tail tips and subjected to Sanger sequencing. Three out of the nine collected embryos had bi-allelic mutations at the PDX1 locus (Fig. 1D). Mutant embryos had large deletions and two of them showed mosaicism. To further examine *PDX1* knockout phenotype in pig embryos, we sectioned the E28 pig embryos and subjected them to histological analyses. Hematoxylin and eosin staining revealed that in wild type embryos, pancreatic progenitor cells are grouped in clusters. In contrast, the pancreatic primordium in all *PDX1* ^−/−^ embryos was highly disorganized (Fig. 1E and data not shown). Immunohistochemical analysis confirmed the lack of PDX1 protein expression in *PDX1* ^−/−^ embryos while the expression of SOX9, the first specific marker and maintenance factor of pancreatic progenitors, was retained, similar to what has been observed in mice^11^. Interestingly, unlike wild type contol, SOX9+ cells in *PDX1* ^−/−^ embryo were disorderly arranged, implicating a failure in further pancreatic development in the absence of PDX1 (Fig. 1E). Taken together, our results showed successful knockout of PDX1 expression in E28 pig embryos by dual-sgRNA/Cas9, which conseqently disrupted normal pancreatic development.

In sum, our study demonstrated dual sgRNA-guided Cas9 nuclease could be sucessfully used to disable pancreatogenesis in both pre- and post-implantation pig embryos. In future studies, combining CRISPR-Cas9 mediated zygote genome editing with chimeric-competent human induced pluripotent stem cells (hiPSCs)^2^ could potentially provide a powerful platform for the generation of human tissues and organs for transplantation.

## Methods

### Animals

All experiments involving animals were performed under the ethical guidelines of the University of California Davis, and approved by the University of California Davis Institutional Animal Care and Use Committee (protocol #18158).

### sgRNA design and *in vitro* transcription

We used the online software (MIT CRISPR Design Tool: http://crispr.mit.edu) to design sgRNAs targeting common sequence of pig and cow *PDX1* gene. The CRISPR/Cas9 target sequences (20 bp target and 3 bp PAM sequence (underlined)) used in this study are shown as follow: sgRNA1, 5′-TCGTACGGGGAGATGTCCGGGGG-3′; gRNA2. 5′-TCACGCGTGGAAAGGCCAGTGGG-3′. The sgRNAs containing T7 promoter were amplified by PCR with the following primers (5′-TAATACGACTCACTATA-G-[19 bp sgRNA target sequence]-GTTTTAGAGCTAGAAATAGC-3′ and 5′-AAAGCACCGACTCGGTGCCACTTTTTCAAGTTGATAACGGACTAGCCTTATTTTAACTTGCTATTTCTAGCTCTAAAAC-3′) using Q5 High-Fidelity DNA Polymerase (NEB) without template DNA. The PCR product was purified using NucleoSpin Gel and PCR Clean-up (MACHEREY-NAGEL). To prepare sgRNA mRNA, the purified PCR product was *in vitro* transcribed by MEGAshortscript T7 Transcription Kit (Invitrogen) following the manufacturer’s instruction. Prepared RNAs were purified using MEGAclear Kit Purification Transcription Reactions (Ambion) and dissolved in water for embryo transfer (Sigma).

### Preparation of DNA from a single blastocyst and genotyping

DNA derived from single blastocysts was prepared for use as a PCR template. Blastocysts were lysed in lysis buffer (Epicentre) and incubated at 65 °C for 6 minutes and 98 °C for 2 minutes. The resulting DNA solution was stored at −20 °C until use. DNA samples were analyzed by performing two rounds of PCR using GoTaq Hot Start Green Master Mix (PROMEGA) with specific primers for *PDX1* sequences in pig (F: 5′-GGCAGTCATGAATGGCGAGGAGCAGTACTACG-3′; R: 5′-CCGGTATGCTTTAGTCCGACTCTGCCTTATCCAC-3′) and in cow (F: TTCAGTGAGGACCCCAGAGTGCTTTCCG-3′: R: 5′- AGCTCTTGGAGACAGGGGCAGTCGAACA-3′). The PCR conditions were 95 °C for 3 min, followed by 35 cycles of 95 °C for 30 sec, 68 °C for 30 sec, 72 °C for 45 sec, and a final step of 72 °C for 5 min. PCR products were subjected to gel electrophoresis and DNA bands were cut and purified using QIAquick Gel extraction kit (QIAGEN). Amplicons were Sanger sequenced by QuintaraBio (http://www.quintarabio.com/services).

### Genotyping of E28 pig embryos

To determine genotypes of gene modified E28 pig embryos, tail tips were used for genomic DNA extraction using DNeasy Blood & Tissue kit (QIAGEN). The genomic DNA sequences including target site were first amplified with PrimeSTAR GXL DNA Polymerase (Takara) with following primers: pPDX1-seqF: 5′-ACAGCTCCAGGTCCCCGGCTCCA-3′ and pPDX1-seqR1: 5′- CCCTACTCGGCTCCCTCGGTGTGCAA-3′. Amplicon: 4271 bp. Then, the PCR product was nested using the following primers and 1st PCR product as a template.

pPDX1-seqF: 5′-GGCAGTCATGAATGGCGAGGAGCAGTACTACG-3′

pPDX1-seqR2: 5′-CCGGTATGCTTTAGTCCGACTCTGCCTTATCCAC-3′

Amplicon 543 bp. PCR products were cloned into the pCR-Blunt II-TOPO vector with Zero Blunt TOPO cloning kit (Invitrogen) and sequenced at least 16 clones. Amplicons were sequenced using an ABI 3730xl sequencer (Applied Biosystems).

### Bovine *in vitro* embryo production

#### Oocyte recovery and *in vitro* maturation (IVM)

Ovaries were collected from a slaughterhouse (Cargill Meat Solutions, Fresno, CA, USA) and transported to the laboratory in insulated container filled with pre-warmed saline solution at ~32 °C. The ovaries were washed several times and placed in a water bath at (37 °C) in saline solution for oocyte aspiration. Oocytes were aspirated from 2 to 6 mm antral follicles using a 21 G butterfly needle connected to a vacuum pump. Cumulus-oocyte complexes (COCs) containing compact and complete cumulus cell layers were selected and matured in groups of 50 COCs in 400 µl of M199 medium supplemented with ALA-glutamine (0.1 mM), Na pyruvate (0.2 mM), gentamicin (5 µg/ml), EGF (50 ng/ml), oFSH (50 ng/ml; National Hormone and Peptide Program), bLH (1 ug/ml; National Hormone and Peptide Program), cysteamine (0.1 mM), and 10% fetal bovine serum (FBS; Hyclone, South Logan, UT, USA). IVM was performed for 22–24 hours in a humidified atmosphere of 5% CO_2_ in air at 38.5 °C.

#### *In vitro* fertilization (IVF)

Fertilization (Day 0) was carried out using frozen-thawed semen. Straws were thawed at 37 °C for 45 seconds and then semen layered onto a 90%/45% Percoll discontinuous density gradient for centrifugation (700 × g for 15 minutes) at room temperature. A second centrifugation (300 × g for 5 minutes) was performed after discarding the supernatant and re-suspending the spermatozoa pellet in TALP-Sperm (pH = 7.4, 295 mOsm)^12, 13^. Matured groups of 15–20 COCs were washed twice and placed in 50 µL of fertilization medium. The final sperm concentration was adjusted to 1 × 10^6^ sperm/ml using a hemocytometer. The fertilization medium was supplemented with BSA (essentially fatty acid free, 6 mg/ml), fructose (90 µg/ml), sodium pyruvate (0.2 mM), MEM Non-essential amino acid solution, gentamicin (0.12 mg/ml) and heparin (20 µg/ml). Oocytes were co-incubated with spermatozoa at 38.5 °C in humidified atmosphere of 5% CO_2_ in air.

#### Embryo culture (IVC)

Presumptive zygotes were mechanically denuded by vortexing for 3–5 minutes in a 1.5 mL tube and 100 uL of SOF-HEPES medium^14^ and cultured in groups of 15–20 in 50 µL drops of potassium simplex optimized medium supplemented with amino acids and 4 mg/mL of BSA (KSOMaa, pH = 7.4, 275 mOsm) (Evolve ZEBV-100, Zenith Biotech, Guilford, CT, USA) for 7 days. On Day 3, 5% FBS was added. Culture conditions were 38.5 °C in a humidified atmosphere of 5% CO_2_, 5% O_2_, and 90% N_2_. Blastocysts were collected on days 7 and 8 post fertilization.

### Porcine parthenogenetic embryo production

#### Oocyte collection and IVM

Oocytes were aspirated from antral follicles (2–4 diameters) of ovaries from prepubertal gilt ovaries collected at a local slaughterhouse (Olson Meat Company, Orland, CA, USA). COCs were washed in TCM-199 (Gibco) containing 0.1% polyvinyl alcohol (PVA), and incubated at 38 °C and 5% CO2 for 48 hours in TCM-199 containing 0.1% PVA, 3.05 mM D-glucose, 0.91 mM sodium pyruvate, 0.5 μg/ml oFSH, 0.5 μg/ml bLH, 10 ng/ml EGF, 10 μg/ml gentamicin (Gibco) and 10% porcine follicle fluid.

#### Parthenogenetic activation

After IVM, maturated oocytes were stripped of their cumulus cells by incubation in 1 mg/ml hyaluronidase and gentle pipetting. Denuded oocytes were washed with MEM containing 25 mM Hepes (Gibco) and electrically activated with two pulses of 120 V/mm for 40 μs, delivered by a BTX Electro Cell Manipulator 2001 (BTX, San Diego, CA, USA) in a 0.5 mm chamber containing 0.3 M mannitol, 0.05 mM CaCl2, 0.1 mM MgSO4 and 0.1% bovine serum albumin (BSA). After washing with PZM-5^15^, the oocytes were incubated in the presence of 5 μg/ml cytochalasin B in PZM-5 for 3 hours to prevent second polar body extrusion and thus generate diploid parthenogenetic embryos.

#### Embryo culture

After activation, porcine zygotes were cultured in the 500 μl of PZM-5^15^ containing 0.3% BSA for 3–5 days. After 4 days of culture, the culture medium was supplemented with 10% FBS (Gemini Bio-Product, CA, USA) at 38.5 °C in a humidified atmosphere of 5% CO_2_, 5% O_2_, and 90% N_2_.

### Microinjection of Cas9 mRNA and sgRNAs to bovine and porcine zygotes

Presumptive zygotes were transferred into a 50 μL drop of SOF-Hepes medium and placed on an inverted microscope (Nikon, Tokyo, Japan) fitted with micromanipulators (Narishige, Tokyo, Japan). Mixture of Cas9 mRNA (Sigma-Aldrich, 25–100 u/g/ml) and sgRNA (25–50 ug/ml) was loaded to a blunt-end micropipette of 5–7 μm internal diameter (ID) connected to a manual hydraulic oil microinjector (Eppendorf, Hamburg, Germany). Zygotes were secured by a holding pipette and a laser (Saturn 5, RI, UK) was used to create a hole in the zona pellucida. The pipette was advanced into the zygote and cytoplasm was aspirated until the plasma membrane was broken to then deliver the Cas9/sgRNA mixture into the cytoplasm. Groups of 15–25 zygotes were manipulated simultaneously and each session was limited to 20 min. After microinjection, the zygotes were returned to culture as described.

### Pig *in vivo* embryo collection and embryo transfer

Estrus and ovulation was induced by intramuscular administration of 400 I.U. equine chorionic gonadotropin and 800 I.U. of human chorionic gonadotropin. (PG600; Merck Animal Heath, Summit, NJ, USA) followed 72 h later by an intramuscular administration of 750 I.U. of hCG (Choluron, Merck Animal Health, Summit, NJ, USA). Estrus was checked twice per day by exposing sows to a mature boar (nose-to-nose contact) and applying manual back pressure. Females that exhibited a standing estrous reflex were used as recipients of surgical embryo transfer 5 to 6 days after hCG administration. Prior to surgical procedure the animals were fasted (food and water) for 24 hours. Anesthesia was induced by intramuscular administration of 2 mg/kg of Telazol (Zoetis, Kalamazoo, MI, USA) prior to intubation and then maintained at a surgical plane by inhalation of Isoflurane (0.5–5% as need to maintain anesthesia). The Ovary and uterus was exposed by a ventral medial laparotomy. The embryos were transferred to the tip of a uterine horn (15 to 20 cm from the uterotubal junction) with the embryo transfer catheter inserted through the uterine wall, which was previously punctured with a blunt needle. Pregnancy was diagnosed by transabdominal ultrasonography (WED-2000AV, Welld, Shenzhen, China) 17 to 20 days after embryo transfer. On day 28 of gestation the animals were deeply anesthetized by intramuscular administration of 2 mg/kg of Telazol (Zoetis, Kalamazoo, MI, USA) and subsequently euthanized by intracardiac administration of 2.25 mL/kg euthanasia solution (Fatal Plus Solution, Vortex Pharmaceutical Ltd, Dearborn, MI, USA). Then, the reproductive tract was removed and transported to the laboratory within 20 min. Once in the laboratory the uterus was opened and fetuses removed from the placenta tissues and individually measured and weighed (Zeiss, Germany).

### Immunocytochemistry

E28 embryos were dissected, immersed in paraformaldehyde and incubated at 4 °C overnight. Then, after overnight cryoprotection in 30% sucrose solution (Sigma), the embryos were embedded in OCT compound (Sakura Finetek) and frozen in dry ice. Embryo sections were cut on a Leica cryostat. The primary antibodies used were rabbit anti-PDX1 (1:1000, Abcam, ab47267) and rabbit anti-SOX9 (1:100, Abcam, ab185230).

## Electronic supplementary material

Supplementary Info
