# Supplementary material for: CRISPR-Cas9 mediated one-step disabling of pancreatogenesis in pigs
Source: Sci Rep. 2017 Sep 5;7:10487. doi: 10.1038/s41598-017-08596-5 (PMC5585254; doi:10.1038/s41598-017-08596-5)
Supplement: Supplementary file 1 — Supplementary Info [file 41598_2017_8596_MOESM1_ESM.pdf]

CRISPR-Cas9 mediated one-step disabling of pancreatogenesis in pigs

Jun Wu[1,2,]*, Marcela Vilarino[3,]*, Keiichiro Suzuki[1,]*, Daiji Okamura[2,4], Yanina Soledad Bogliotti[3], Insung Park[3], Joan Rowe[5], Bret McNabb[5], Pablo Juan Ross[3,§], Juan Carlos Izpisua Belmonte[1,§]

* These authors contributed equally to this work

§ Correspondence: Pablo Juan Ross, pross@ucdavis.edu

Juan Carlos Izpisua Belmonte, belmonte@salk.edu

Affiliations:

1. Salk Institute for Biological Studies, 10010 N. Torrey Pines Rd., La Jolla, CA 92037 USA
2. Universidad Católica San Antonio de Murcia, Campus de los Jerónimos 135, Guadalupe, 30107, Spain
3. Department of Animal Science, University of California Davis, One Shields Avenue, Davis, CA 95616
4. Department of Advanced Bioscience, Graduate School of Agriculture, Kindai University, 3327-204 Nakamachi, Nara 631-8505, Japan.
5. School of Veterinary Medicine, University of California Davis, One Shields Avenue, Davis, CA 95616

A

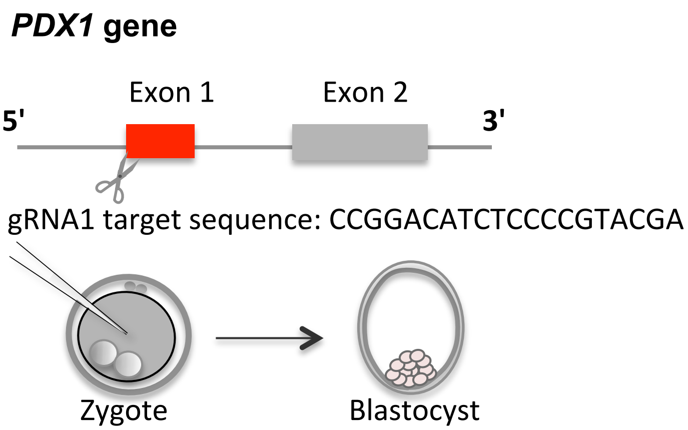

B

Bovine *PDX1* locus

gRNA target site

```
5’GCTGGAGCAGGGCAGCCCCCCGGACATCTCCCCGTACGAGGTGCCTCCGCTAGCCG-3’
```

Mono-allelic mutations

```
5’-GCTGGAGCAGGGCAGCCCCCC------TCCCCGTACGAGGTGCCTCCGCTAGCCG-3’ (-6)
5’-GCTGGAGCAGGGCAGCCCCCC------TCCCCGTACGAGGTGCCTCCGCTAGCCG-3’ (-6)
5’-CCCTGGCCTG-------------------------------------GGCTGG-3’ (+4, -124)
5’-GCTGGAGCAGGGCAGCCCCCC------TCCCCGTACGAGGTGCCTCCGCTAGCCG-3’ (-6)
5’-GCTGGAGCAGGGCAGCCCCC-----------TGGTACGAGGTGCCTCCGCTAGCCG-3’(+2, -13)
```

Bi-allelic mutations

```
5’-GCTGGAGCAGGGCAGCCCCCCGTACATCTCCCCGTACGAGGTGCCTCCGCTA-3’     (1 sub.)
5’-GCTGGAGCAGGGCAGCCCCCCG------------TACGAGGTGCCTCCGCTAGC-3’   (-12)
5’-GCTGGAGCAGGGCAGCCCCCC------------TACGAGGTGCCTCCGCTA-3’      (-12)
5’-TCCCCG---------------------------TACGAGGTGCCTCCGCTAGC-3’    (-47)
5’-GCTGGAGCAGGGCAGCCCCTCCCGTGCACCTCGTACGAGGTGCCTCCGCTAGC-3’    (12 sub.)
5’-GCTGGAGCAGGGCAGCCCCCCGCACATCTCCCCGTACGAGGTGCCTCCGCTAGC-3’   (+1)
5’-GCTGGAGCAGGGCAGCCCCCC----------------------------TGGAGG-3’ (-137)
5’-GCTGGAGCAGGGCAGCCCCCC----------------------------TGGAGG-3’ (-12)
5’-GCTGGAGCAGG------------ACATCTCCCCGTACGAGGTGCCTCCGCTAGCCG-3’(-72)
5’-GCTGGAGCAGGGCAGCCCCCC------------TACGAGGTGCCTCCGCTAGCCG-3’ (-12)
5’-GCTGGAGCAGGGCAGCCCCCC------------TACGAGGTGCCTCCGCTAGCCG-3’ (-12)
```

WT

```
5’GCTGGAGCAGGGCAGCCCCCCGGACATCTCCCCGTACGAGGTGCCTCCGCTAGCCG-3’
5’GCTGGAGCAGGGCAGCCCCCCGGACATCTCCCCGTACGAGGTGCCTCCGCTAGCCG-3’
```

C

Pig *PDX1* locus

gRNA target site

```
5’-GCGCGCTGGAGCAGGGCGGCCCCCCGGACATCTCCCCGTACGAGGTGCCCCCGCTCGCCGACG-3’
```

Mono-allelic mutations

```
5’-GCGCGCTGGAGCAGGGCGACCCCCCGGACATCTCCCCGTACGAGGTGCCCCCGCTCCCCGACG-3’ (2 sub.)
5’-TGGGCCCCCGCGACCAGTG-------------GGCCCCCCCGGACATCTCCCCGTACGAGGTG-3’ (+19, -48)
```

WT

```
5’-GCGCGCTGGAGCAGGGCGGCCCCCCGGACATCTCCCCGTACGAGGTGCCCCCGCTCGCCGACG-3’
5’-GCGCGCTGGAGCAGGGCGGCCCCCCGGACATCTCCCTGTACGAGGTGCCCCCGCTCGCCGATG-3’
5’-GCGCGCTGGAGCAGGGCGGCCCCCCGGACATCTCCCCGTACGAGGTGCCCCCGCTCGCCGACG-3’
5’-GCGCGCTGGAGCAGGGCGGCCCCCCGGACATCTCCCCGTACGAGGTGCCCCCGCTCGCCGACG-3’
5’-GCGCGCTGGAGCAGGGCGGCCCCCCGGACATCTCCCCGTACGAGGTGCCCCCGCTCGCCGACG-3’
5’-GCGCGCTGGAGCAGGGCGGCCCCCCGGACATCTCCCCGTACGAGGTGCCCCCGCTCGCCGACG-3’
5’-GCGCGCTGGAGCAGGGCGGCCCCCCGGACATCTCCCCGTACGAGGTGCCCCCGCTCGCCGACG-3’
5’-GCGCGCTGGAGCAGGGCGGCCCCCCGGACATCTCCCCGTACGAGGTGCCCCCGCTCGCCGACG-3’
5’-GCGCGCTGGAGCAGGGCGGCCCCCCGGACATCTCCCCGTACGAGGTGCCCCCGCTCGCCGACG-3’
5’-GCGCGCTGGAGCAGGGCGGCCCCCCGGACATCTCCCCGTACGAGGTGCCCCCGCTCGCCGACG-3’
5’-GCGCGCTGGAGCAGGGCGGCCCCCCGGACATCTCCCCGTACGAGGTGCCCCCGCTCGCCGACG-3’
```

A

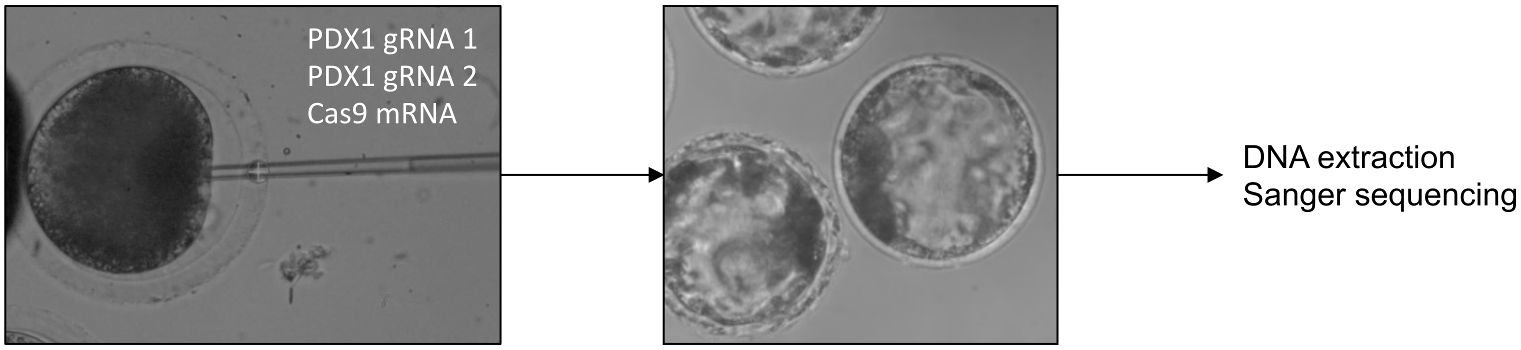

B

Pig *PDX1* locus

gRNA1 target site (reverse) **gRNA2** target site (forward)

```
5'-GGCGGCCCCCCGGACATCTCCCCGTACGAGGTGCCC-------158bp-------CAAGGCTCACGCGTGGAAAGGCCAGTGGGCAG-3'
```

Mono-allelic mutations

```
5'---//--CCCGTACGAGGTGCCCCCCGTACGAGGTGCCCCCGCTCGCCGTG--------//---------GGAAAGGCCAGTGGGCAG-3'   (-37, +2, -165)
5'-GGCGGCCCTTA----------//----------------------------------------------------CCTGTGGGCAG-3'   (+3, -212)
5'-GGCGGCCCCCCGGACAACTCCCAGCACGAAGAGCCACCG[]------------------//------------------GGGCAG-3'   (+187, -184)
5'-GTA-------------------------------------------------------//------------------AGTGGGCAG-3'   (+3, -223)
5'-GGCGGCCCCCCGGACATCTCCCCGTACGAGGTGCCC[]//[]------//-------------------------CCAGTGGGCAG-3'   (+10, -11, +38, - 39)
5'------------------------------------------//------------------------------------------3'   (-298)
5'-----------------------------------------//-------------------------------------------3'   (-254)
5'-GGCGGCCCCCCG----------//-----------------------------------------------------AGTGGGCAG-3'   (-208)
5'-[]---------------------------//----------------------------------------------AGTGGGCAG-3'   (+72, -265)
5'-------------------------------//---------------------------------------------GTGGGCAG-3'   (-243)
```

Bi-allelic mutations

```
5'- ---------------------------------------//-------------------------------GGGCAGTGGGCAG-3'   (-255)
5'-GGCGGCCCCCCG---------------------//---------------------------------------AGTGGGCAG-3'   (-208)
5'- ---------------------------------------//------------------------------------AGTGGGCAG-3'   (-242)
```

Wild-type

```
5'-GGCGGCCCCCCGGACATCTCCCCGTACGAGGTGCCC-------158bp-------CAAGGCTCACGCGTGGAAAGGCCAGTGGGCAG-3'
5'-GGCGGCCCCCCGGACATCTCCCCGTACGAGGTGCCC-------158bp-------CAAGGCTCACGCGTGGAAAGGCCAGTGGGCAG-3'
5'-GGCGGCCCCCCGGACATCTCCCCGTACGAGGTGCCC-------158bp-------CAAGGCTCACGCGTGGAAAGGCCAGTGGGCAG-3'
5'-GGCGGCCCCCCGGACATCTCCCCGTACGAGGTGCCC-------158bp-------CAAGGCTCACGCGTGGAAAGGCCAGTGGGCAG-3'
5'-GGCGGCCCCCCGGACATCTCCCCGTACGAGGTGCCC-------158bp-------CAAGGCTCACGCGTGGAAAGGCCAGTGGGCAG-3'
5'-GGCGGCCCCCCGGACATCTCCCCGTACGAGGTGCCC-------158bp-------CAAGGCTCACGCGTGGAAAGGCCAGTGGGCAG-3'
5'-GGCGGCCCCCCGGACATCTCCCCGTACGAGGTGCCC-------158bp-------CAAGGCTCACGCGTGGAAAGGCCAGTGGGCAG-3'
5'-GGCGGCCCCCCGGACATCTCCCCGTACGAGGTGCCC-------158bp-------CAAGGCTCACGCGTGGAAAGGCCAGTGGGCAG-3'
5'-GGCGGCCCCCCGGACATCTCCCCGTACGAGGTGCCC-------158bp-------CAAGGCTCACGCGTGGAAAGGCCAGTGGGCAG-3'
```

C

E28 pig embryos

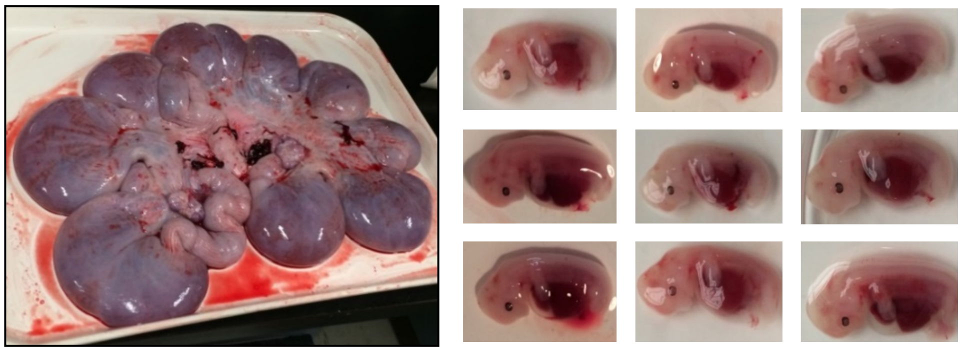

Supplementary figure legends

Figure S1. Gene disruption with single sgRNA-guided Cas9 at pig and cattle *PDX1* loci. (A) Schematic diagram showing the location and sequence of a gRNA (gRNA1) designed to target exon1 of both pig and cattle *PDX1* gene. Bovine and porcine zygotes were co-injected with Cas9 mRNA and sgRNA1 and *in vitro* cultured until the blastocyst stage for blastocyst genotyping. (B) Sanger sequencing results for bovine blastocysts. The results were from two independent experiments (C) Sanger sequencing results for porcine blastocysts. The results were from two independent experiments. Red dashes represent deletions and red letters indicate insertions or substitutions; insertions (+) or deletions (-) are shown to the right of each allele. For cattle, the mutated blastocysts (83.3%, 10/12) are composed of 41.7% (5/12) of mono-allelic mutations and 41.7% (5/12) of bi-allelic mutations. For pigs they were 15% (2/13) of the embryos with mono-allelic mutations.

Figure S2. Gene disruption with dual sgRNA-guided Cas9 at pig *PDX1* loci. (A) The workflow in testing the efficacy and efficiency of *PDX1* disruption with dual sgRNA-guided Cas9. Cas9 mRNA and PDX1 sgRNA1 and sgRNA2 were co-injected into a pig zygote and cultured until the blastocyst stage. Genomic DNA was isolated from the blastocysts and subjected to Sanger sequencing to determine the genotypes. (B) Sanger sequencing results at the *PDX1* locus of porcine blastocysts developed from zygotes injected with two sgRNAs. The results were from two independent experiments. The PAM sequence is underlined, and the targeted sequences complementary to PDX1 gRNA1 and gRNA2 are shown in blue. Red dashes and // represent deletions and red letters and [] insertions; insertions (+) or deletions (-) are shown to the right of each allele. (C) Left, a representative bright field image showing the porcine female reproductive tract at day 28 of pregnancy. Right, nine pig embryos were collected at day 28 of development.

Full gel image for Figure 1B

Pig embryos injected with PDX1 gRNAs (g1/g2)

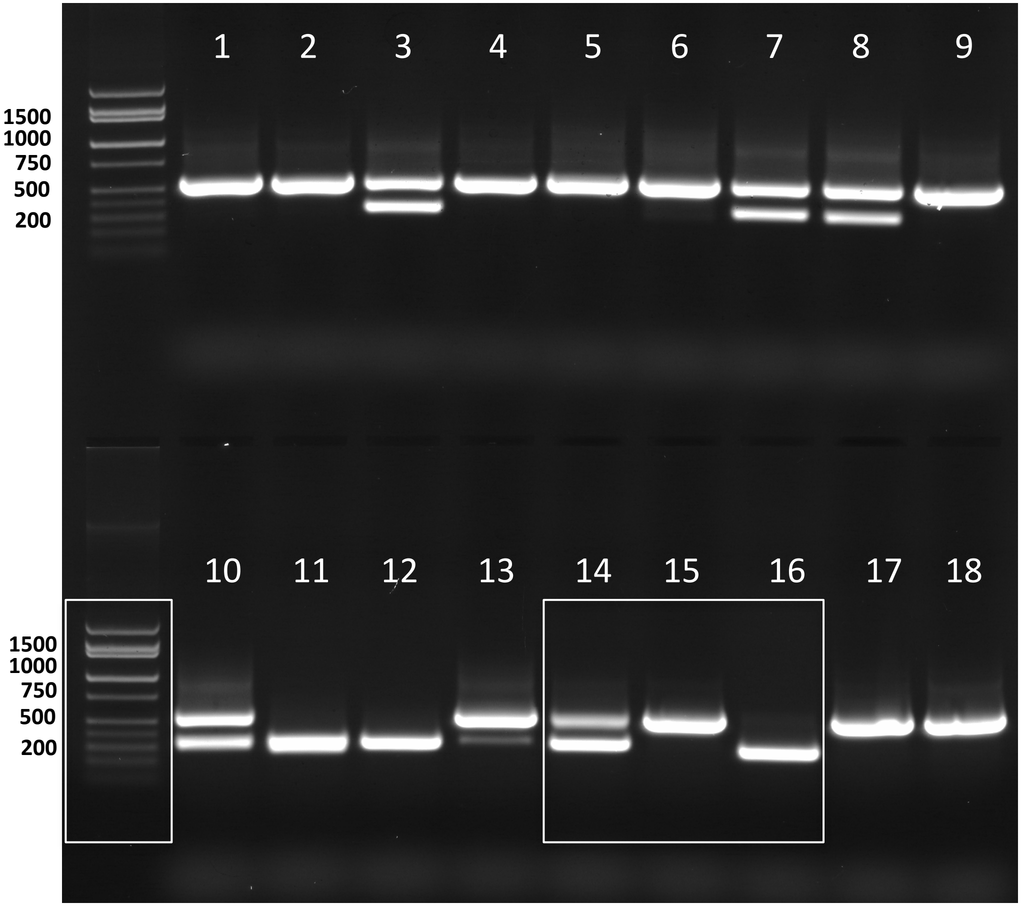

1-2 & 4-5: PDX1-g1
3 & 6-17: PDX1-g1+g2
18: Control pig genomic DNA
